# Impending Catastrophe of Delayed Fracture Management During the COVID-19 Pandemic

**DOI:** 10.1017/dmp.2020.273

**Published:** 2020-07-27

**Authors:** Michael Anthonius Lim, Raymond Pranata

**Affiliations:** Faculty of Medicine, Universitas Pelita Harapan, Tangerang, Indonesia

**Keywords:** COVID-19, fracture, manipulation, massage, trauma

The coronavirus disease (COVID-19) pandemic has led to a drastic reduction in the number of road-traffic accidents, sports injuries, and eventual orthopedic surgeries. During such quiet situations, where lockdown and social distancing are widely implemented, many people struggled to access specialized medical services in health care facilities, especially given the surge in the number of patients and prioritization of COVID-19 patients and emergency cases. Many people consider orthopedic management to be a last resort and often opt instead for more traditional, but unproven, medical treatments. Traditional massage and manipulation or alternative medicine continue to be popular, especially in Eastern countries. Traumatic cases, such as fractures, dislocations, ligament tears, and tendon ruptures, are often treated by unregistered practitioners of such alternative and traditional treatments. In many remote areas, the community of patients relies heavily on traditional healing using herbs, potions, massages, and, in some cases, ancient, mystical, and magical rituals passed down from their ancestors or learned through religious teachings.

The reluctance of people to seek orthopedic services for their injury management can result in adverse outcomes in both the short and long term. If any fractures are not managed immediately, early complications can arise, such as compartment syndrome, nerve compression, vascular damage, hemorrhagic shock, bone and soft tissue infections (eg, osteomyelitis), and deep vein thrombosis. Long-term complications include delayed union, nonunion, malunion, avascular bone necrosis, myositis ossificans, and disuse osteoporosis. Immobilization of a limb and its joints leads to progressive structural, metabolic, and biomechanical changes. Muscle atrophy starts within a few days of non-weight bearing, along with the atrophy of the bone, ligament, synovium, and articular cartilage, followed by reduced muscular activity, degenerative changes to the bone and joint, and last, permanent changes due to prolonged immobilization. Complications of inadequately managed fractures can have a significant influence on the patient’s well-being, recovery, and quality of life.

A soft tissue massage or manipulation can be helpful, rather than harmful, as it relieves pain (releases tension on the peripheral nerve endings) and reduces swelling and effusions (aids the onward flow in the lymphatic system and superficial veins), and eventually restores muscle tone and function. However, the performance of such maneuvers performed by nonprofessional practitioners and the use of forceful techniques are often associated with adverse outcomes, including soft tissue trauma, neurologic compromise, disk herniation, spinal cord injury, bone fracture, and dislocation.^1^ Such nonprofessional therapists, who generally do not receive professional medical education or training, claim to be capable in diagnosing and treating fractures and any injuries thoroughly, and often request for radiographic imaging of the injured area. Daily fracture massages performed by the traditional bone setters create a macro movement at the fracture site and contribute to nonunion or malunion.^2^ These therapists, through the use of inadequate personal protective equipment, may contract COVID-19 through contact with infected patients and, in turn, may cause transmission of the disease to other patients. This is dangerous, especially for older patients and those with comorbidities who have an increased risk of mortality.^3-5^ Complications associated with maneuvers or delayed/inadequate treatment coupled with COVID-19 in vulnerable patients may prove detrimental.

Considering the uncertainties during the COVID-19 pandemic and the tendency to seek alternative traditional treatments for fractures and other traumatic injuries in an attempt to avoid hospital visits, we will undoubtedly see an increase in the number of serious musculoskeletal complications in the future. Increased inflammation and hematoma lead to impending compartment syndrome, which requires emergency fasciotomy, while the presence of bacterial contamination and extensive soft tissue trauma predispose the patient to the development of acute and chronic osteomyelitis, which requires the prompt elimination of the infection (debridement, drainage, dead space removal, and long-term antibiotics) and stabilization of the fracture. Finally, the possibility of increasing incidences of delayed union, malunion, and nonunion is inevitable.

## References

[1] Yin P , Gao N , Wu J , et al. Adverse events of massage therapy in pain-related conditions: a systematic review. Evid Based Complement Alternat Med. 2014;2014:480956. doi: 10.1155/2014/480956. 25197310 PMC4145795

[2] Ogunlade SO , Omololu AB , Alonge TO , et al. Predisposing factors and outcome of treatment of non-union of long-bone fractures in Ibadan, Nigeria. Niger Postgrad Med J. 2011;18(1):56-60. 21445115

[3] Pranata R , Lim MA , Huang I , et al. Hypertension is associated with increased mortality and severity of disease in COVID-19 pneumonia: a systematic review, meta-analysis and meta-regression. J Renin Angiotensin Aldosterone Syst. 2020;21(2):1-11. doi: 10.1177/14703203209268. PMC7231906 32408793

[4] Huang I , Lim MA , Pranata R. Diabetes mellitus is associated with increased mortality and severity of disease in COVID-19 pneumonia – a systematic review, meta-analysis, and meta-regression: diabetes and COVID-19. Diabetes Metab Syndr Clin Res Rev. 2020;14(4):395-403. doi: 10.1016/j.dsx.2020.04.018. PMC7162793 32334395

[5] Pranata R , Huang I , Lim MA , et al. Impact of cerebrovascular and cardiovascular diseases on mortality and severity of COVID-19 – systematic review, meta-analysis, and meta-regression. J Stroke Cerebrovasc Dis. 2020;epub, doi: 10.1016/j.jstrokecerebrovasdis.2020.104949. PMC7221373 32410807

